# Negative-Pressure Cavitation Extraction of Secoisolariciresinol Diglycoside from Flaxseed Cakes

**DOI:** 10.3390/molecules200611076

**Published:** 2015-06-15

**Authors:** Hao Tian, Wan-Yi Li, Dan Xiao, Zhi-Min Li, Jian-Wen Wang

**Affiliations:** 1 College of Pharmaceutical Sciences, Soochow University, Suzhou 215123, China; E-Mail: tianhao.630@163.com; 2 Institute of Medicinal Plants, Yunnan Academy of Agricultural Sciences, Kunming 650205, China; E-Mails: xiaodanms@126.com; (D.X.); lzmyaas@163.com (Z.-M.L.)

**Keywords:** flaxseed cake, negative-pressure cavitation extraction, pilot-scale application, response surface methodology, secoisolariciresinol diglucoside

## Abstract

The negative-pressure cavitation extraction (NPCE) technique was applied firstly to extract secoisolariciresinol diglucoside (SDG) from flaxseed cakes. The significant extraction parameters were screened by fractional factorial design (FFD). The optimal parameters were determined using the central composite design (CCD) with the two variables, NaOH amount and the liquid/solid ratio. The conditions of the extraction were optimized by using response surface methodology (RSM). Under the optimal conditions, the extraction yield and the extraction purity of SDG was 16.25 mg/g and 3.86%, respectively. The efficiency of NPCE was compared with that of conventional extraction methods. Our results demonstrated that NPCE was comparable to the well-known ultrasound-assisted extraction in term of extraction yield and purity. This extraction technique has advantages of less time-consuming, low solvent usage and high throughput capability.

## 1. Introduction

Flax (*Linum usitatissimum* L.) is an economically important oilseed and fiber crop. Traditionally, flaxseed oil (40%–45%, *w*/*w*) has been used mostly for industrial purposes in the manufacture of paints, varnishes, soaps and printer inks [1]. Recently, flaxseed oil rich in α-linolenic acid has been marketed as a health food to minimize disease risks associated with hyperlipidemia, mammary cancer and atherosclerosis [2,3,4]. After the oil is extracted from the seed, the flaxseed cake is used usually for livestock feed as a by-product. However, bioactive lignans are present in flaxseed cakes in a higher concentration than in other plant sources. The main lignan in flaxseed meal is secoisolariciresinol diglucoside (SDG) in concentrations varied from 6–18 mg/g dry weight (DW) [5,6]. SDG was found to be a precursor of mammalian phytoestrogens enterodiol and enterolactone, which have estrogenic activity and play an important role in the prevention of breast and prostate cancer incidence [7]. SDG has gained significant attention in nutritional and functional foods due to its substantial biological activities including potential antioxidant activity, retardation of hypercholesterolemic atherosclerosis and preventing development of type 1 and 2 diabetes [8,9,10,11]. The conventional SDG extraction methods involve a grinding pretreatment, a solvent extraction of the lignan polymers with aqueous ethanol, methanol or acetone and alkaline hydrolysis, releasing SDG by cleavage of the ester linkages in the SDG-3-hydroxy-3-methylglutaric acid (HMGA) oligomer [12,13,14]. Recently, the concern about environmentally friendly, selective, and effective techniques for lignan extraction from flaxseed has gained in interest. Among these techniques, microwaves, enzyme (β-glucuronidase/sulfatase or cellulase) aided extraction and electrotechnologies such as high voltage electrical discharge and pulsed electric fields have shown their efficiencies for SDG extraction from flaxseed [15,16,17,18,19].

A novel, environmentally friendly, and efficient extraction technique using negative pressure cavitation (NPC) has been successfully applied to extract phytochemicals including flavonoids, isoflavonoids, triterpenoid saponins, stilbenes, polyphenols, alkaloids and polysaccharides [20,21,22,23,24,25,26]. In the NPC system, cavitation is formed under negative pressure created by a vacuum pump to corrode the surface of solid particles. The turbulence, collision and mass transfer between the extraction solvent and matrix are intensified when air is introduced continuously into the system through the valet [27,28]. Negative-pressure cavitation extraction (NPCE) has been mainly used on dried roots and leaves [23,25,26,29]. However, this promising technology has never been used for seeds or the seed hulls.

In the present study, NPCE was applied to extract SDG from flaxseed cakes and compared with other conventional solid–liquid extraction methods. The effect of extraction pressure, temperature, solvent, liquid-solid ratio, extraction time, soaking time and ventilation volume will be investigated using response surface methodology (RSM). This study aimed at optimizing the combination of factors in order to reach the highest SDG extraction yield and purity, and proposing an efficient alternative to the classical solvent extraction of flaxseed lignans.

## 2. Results and Discussion

### 2.1. Significant Parameters Screened by Fractional Factorial Design (FFD)

The effects of the following parameters on NPCE efficiency were determined on the basis of preliminary experiments: extraction pressure, temperature, ethanol concentration, NaOH amount, liquid/solid ratio, extraction time, ventilation volume and soaking time. The results of the fractional factorial design were shown in Table 1. Regression analysis resulted in the following functions, which described SDG yield (*Y*_1_, mg/g) and purity (*Y*_2_, %) as functions of the coded levels of all parameters:

*Y*_1_ = 8.96 − 0.33*X*_1_ + 1.85*X*_2_ − 2.28*X*_3_ + 3.23*X*_4_ + 2.06*X*_5_ + 1.03*X*_6_ + 0.19*X*_7_ + 0.85*X*_8_ (1)

*Y*_2_ = 2.38 − 0.19*X*_1_ + 0.42*X*_2_ + 0.23*X*_3_ + 0.69*X*_4_ + 0.66*X*_5_ + 0.03*X*_6_ − 0.02*X*_7_ + 0.15*X*_8_ (2)

**Table 1:** Levels of the variables tested in the 2^(9−5)^ experimental design and statistics analysis of secoisolariciresinol diglucoside (SDG) yield and purity.

Run	Factors																					Results			
	*X*_1_^a^ (Mpa)		*X*_2_^b^ (°C)			*X*_3_^c^ (%)		*X*_4_^d^ (%)			*X*_5_^e^ (mL/g)			*X*_6_^f^ (min)		*X*_7_^g^ (L/h)			*X*_8_^h^ (h)		*X*_9_	SDG Yield (mg/g DW)		SDG Purity (%)	
6	−0.06(+1)		20(−1)		90(+1)		0.3(−1)			6(−1)			60(+1)		20(−1)			4(+1)			+1	0.07		0.05	
7	−0.02(−1)		50(+1)		90(+1)		0.3(−1)			6(−1)			10(−1)		160(+1)			4(+1)			+1	2.04		1.06	
12	−0.06(+1)		50(+1)		40(−1)		2.0(+1)			6(−1)			10(−1)		20(−1)			4(+1)			−1	13.56		2.75	
20(C)	−0.04(0)		35(0)		65(0)		1.15(0)			13(0)			35(0)		90(0)			2(0)			0	15.17		3.09	
15	−0.02(−1)		50(+1)		90(+1)		2.0(+1)			6(−1)			60(+1)		20(−1)			0(−1)			−1	7.36		2.75	
3	−0.02(−1)		50(+1)		40(−1)		0.3(−1)			20(+1)			60(+1)		20(−1)			4(+1)			−1	11.96		2.77	
10	−0.06(+1)		20(−1)		40(−1)		2.0(+1)			20(+1)			60(+1)		20(−1)			0(−1)			+1	14.11		2.66	
14	−0.06(+1)		20(−1)		90(+1)		2.0(+1)			6(−1)			10(−1)		160(+1)			0(−1)			−1	3.32		2.43	
8	−0.06(+1)		50(+1)		90(+1)		0.3(−1)			20(+1)			10(−1)		20(−1)			0(−1)			−1	5.17		2.78	
18(C)	−0.04(0)		35(0)		65(0)		1.15(0)			13(0)			35(0)		90(0)			2(0)			0	15.38		3.47	
2	−0.06(+1)		20(−1)		40(−1)		0.3(−1)			20(+1)			10(−1)		160(+1)			4(+1)			−1	6.56		1.94	
19(C)	−0.04(0)		35(0)		65(0)		1.15(0)			13(0)			35(0)		90(0)			2(0)			0	15.66		3.53	
1	−0.02(−1)		20(−1)		40(−1)		0.3(−1)			6(−1)			10(−1)		20(−1)			0(−1)			+1	0.25		0.08	
13	−0.02(−1)		20(−1)		90(+1)		2.0(+1)			20(+1)			10(−1)		20(−1)			4(+1)			+1	4.80		3.54	
17(C)	−0.04(0)		35(0)		65(0)		1.15(0)			13(0)			35(0)		90(0)			2(0)			0	15.23		3.20	
16	−0.06(+1)		50(+1)		90(+1)		2.0(+1)			20(+1)			60(+1)		160(+1)			4(+1)			+1	14.79		4.80	
4	−0.06(+1)		50(+1)		40(−1)		0.3)−1)			6(−1)			60(+1)		160(+1)			0(−1)			+1	3.88		1.30	
9	−0.02(−1)		20(−1)		40(−1)		2.0(+1)			6(−1)			60(+1)		160(+1)			4(+1)			−1	11.88		1.48	
11	−0.02(−1)		50(+1)		40(−1)		2.0(+1)			20(+1)			10(−1)		160(+1)			0(−1)			+1	14.89		2.34	
5	−0.02(−1)		20(−1)		90(+1)		0.3(−1)			20(+1)			60(+1)		160(+1)			0(−1)			−1	3.03		1.66	
**Factor**		**Statistics Analysis**																							
		**Effect**							**Coefficient**								**t(11)**						***p***		
		**Yield**		**Purity**					**Yield**			**Purity**					**Yield**			**Purity**			**Yield**		**Purity**
Mean/Intercept		8.95556		2.383200					8.95556			2.383200					8.76743			13.06841			0.000003		0.000000
*X_1_*		−0.65728		−0.380250					−0.32864			−0.190125					−0.28777			−0.93250			0.778873		0.371096
*X_2_*		3.70416		0.839750					1.85208			0.419875					1.62175			2.05934			0.133143		0.063928
*X_3_*		−4.56276		0.470000					−2.28138			0.235000					−1.99766			1.15259			0.071088		0.273511
*X_4_*		6.46816		1.390750					3.23408			0.695375					2.83188			3.41057			0.016317		0.005819
*X_5_*		4.12033		1.324500					2.06017			0.662250					1.80396			3.24810			0.098662		0.007761
*X_6_*		2.06056		0.066000					1.03028			0.033000					0.90215			0.16185			0.386303		0.874356
*X_7_*		0.38872		−0.045500					0.19436			−0.022750					0.17019			−0.11158			0.867952		0.913166
*X_8_*		1.70805		0.299250					0.85404			0.149625					0.74782			0.73386			0.470256		0.478386

+1 High level; −1 Low level; 0 Center point; ^a^ Negative pressure (MPa); ^b^ Extraction temperature (°C); ^c^ Ethanol concentration (%); ^d^ Amount of NaOH (%); ^e^ Liquid/solid ratio (mL/g); ^f^ Extraction time (min); ^g^ Ventilation volume (L/h); ^h^ Soaking time (h).

In this experiment, statistical analysis of the data (*t*-test) (Table 1) showed that only the amount of NaOH (*X*_4_) and liquid/solid ratio (*X*_5_) had a significant effect (*p* < 0.05). As the major part of SDG retained in the flaxseed cake is ester-linked to HMGA to form SDG–HMGA oligomers, the release of SDG from SDG–HMGA oligomers is generally achieved by alcoholic solid–liquid extraction and alkaline treatment [30,31]. Eliasson *et al.* found the direct alkaline hydrolysis led to a higher SDG yield from defatted flaxseed flour when compared with the alkaline hydrolysis after dioxane–ethanol extraction [14]. In present study, the direct alkaline hydrolysis method was adopted in the NPCE system. The formation, expansion and collapse of tiny bubbles under negative pressure occurred in NPCE [23]. In the area of bubble formation, the negative pressure and surface tension interrupted airflow to generate tiny bubbles, resulting in a highly instable liquid-gas-solid system. Subsequently, during the collapse of bubbles in the suspension area, intensive cavitation and stirring effects could make solvent diffuse easily into the inside of material. SDG–HMGA oligomers in the matrix were hydrolyzed in alkaline solution and SDG were released efficiently into the solvent due to an intense stirring and mass transferring. Hence, the effect of alkali concentration used in NPCE was significant (Table 1). In general, increasing the liquid/solid ratio could enhance the yield of extracted compound. Ho *et al.* applied pressurized low polarity water to extract lignan from defatted flaxseed meal. They found that SDG yield increased linearly with liquid-solid ratio until the temperature-liquid/solid ratio interaction became dominant at about 160 °C [32].

Although the significant parameters could be screened by FFD, it cannot predict the optimal levels of those parameters that significantly affect SDG production. Hence, the central composite design (CCD) was used in the following optimization steps.

### 2.2. Significant Parameters Optimized by CCD

The optimal parameters were determined using CCD with the two variables, NaOH amount and the liquid/solid ratio. The levels of the two variable factors and experimental results are shown in Table 2. The fitted equation for estimation of SDG yield and purity had the following form:

*Y*_1_ = 16.59 + 0.03*X*_4_ − 0.05*X*_4_^2^ + 0.03*X*_5_ − 0.06*X*_5_^2^ − 0.02*X*_4_*X*_5_ (3)

*Y*_2_ = 3.97 + 0.02*X*_4_ − 0.23*X*_4_^2^ − 0.18*X*_5_ − 0.22*X*_5_^2^ + 0.02*X*_4_*X*_5_ (4)

where *Y*_1_ is the yield of SDG (mg/g), and *Y*_2_ is purity (%).

**Figure 1:**
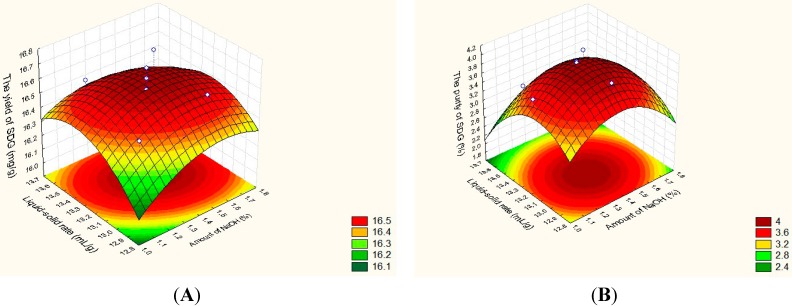
The response surfaces representations for SDG yield (**A**) and purity (**B**) in flaxseed cakes.

According to the quadratic coefficients, the effects of the independent factors on the index of investigation were shown in the following icons. The two parabolas have maximum points, respectively, in the similar investigation region, which were illustrated in Figure 1. Taking into account the yield and the purity of SDG, from the Equations (3) and (4), the maximum point was 1.39% for NaOH amount, 13.16:1 for liquid/solid rate (mL/g), respectively. The optimized response was predicted and at this point, SDG yield and extraction purity was predicted at the response of 16.60 mg/g and 4.00%, respectively.

Thus, the experiments showed that NaOH amount and the liquid/solid ratio were the major influencing factors. At last, from the economic efficiency, the optimizing process for the extraction of SDG from flaxseed cakes by NPCE included: extraction pressure −0.04 MPa, extraction temperature 35 °C, concentration of ethanol 65% (*v*/*v*), extraction time 35 min, ventilation volume 90 L/h, soaking time 0 h, the amount of NaOH 1.39% and the liquid/solid rate (mL/g) 13.16:1.

**Table 2:** The experiment design and results of central composite design (CCD) and the statistics analysis of SDG yield and purity.

Run	Factors			Yield (mg/g DW)				Purity (%)	
	Block	*X*_4_ (%)	*X*_5_ (mL/g)	Observed		Predicted		Observed	Predicted
1	1	1.150(−1)	13.000(−1)	16.44		16.40		3.48	3.70
2	1	1.150(−1)	13.480(+1)	16.62		16.50		3.42	3.31
3	1	1.600(+1)	13.000(−1)	16.55		16.51		3.64	3.69
4	1	1.600(+1)	13.480(+1)	16.64		16.53		3.66	3.38
5	1	1.375(0)	13.240(0)	16.41		16.59		3.96	3.97
6	1	1.375(0)	13.240(0)	16.65		16.59		3.97	3.97
7	2	1.057(−1.414)	13.240(0)	16.36		16.45		3.58	3.50
8	2	1.693(1.414)	13.240(0)	16.47		16.55		3.39	3.54
9	2	1.375(0)	12.901(−1.414)	16.40		16.43		3.98	3.77
10	2	1.375(0)	13.579(1.414)	16.38		16.51		3.00	3.27
11	2	1.375(0)	13.240(0)	16.57		16.59		3.99	3.97
12	2	1.375(0)	13.240(0)	16.72		16.59		3.96	3.97
**Factor**	**Statistics Analysis**								
	**Effect**		**Coefficient**		**t(5)**			***p***	
	**Yield**	**Purity**	**Yield**	**Purity**	**Yield**		**Purity**	**Yield**	**Purity**
Mean/Intercept	16.58964	3.970503	16.58964	3.970503	230.3988		33.38118	0.000000	0.000000
(1)*X*_4_(L)	0.06809	0.031545	0.03405	0.015772	0.6687		0.18753	0.518951	0.858618
*X*_4_(Q)	−0.09305	−0.454056	−0.04653	−0.227028	−0.8173		−2.41433	0.434551	0.060540
(2)*X*_5_(L)	0.06097	−0.354486	0.03048	−0.177243	0.5987		−2.10737	0.562198	0.088918
X_5_(Q)	−0.11901	−0.449047	−0.05951	−0.224523	−1.0454		−2.38769	0.325443	0.062566
1L by 2L	−0.04456	0.041881	−0.02228	0.020940	−0.3094		0.17605	0.762011	0.867161

### 2.3. Model Validation and NPCE Pilot-Scale Application

The verification experiments were carried out under above optimized parameters. Under these conditions, the extraction yield of SDG and purity reached 16.25 ± 0.37 mg/g, 3.86% ± 0.21% (*n* = 3), whereas the response value predicated by the model equations was 16.60 mg/g and 4.00%, respectively. The results indicated the experimental values were in good agreement with the predicted ones, suggesting the reliable RSM models used in NPCE.

The experiment of NPCE pilot-scale application was carried out under the optimized conditions in above study. The extraction yield of SDG and purity obtained using pilot-scale was 15.61 ± 0.84 mg/g, 3.67% ± 0.30%, respectively, which was close to the NPCE results in laboratory scale (16.25 ± 0.37 mg/g, 3.86% ± 0.21%). These indicated that NPCE process for SDG yield was scalable to pilot-scale application.

### 2.4. Comparison of NPCE with Other Extraction Methods

Although NPCE has received considerable attention for the extraction of bioactive compounds from dry leaves and roots, NPCE of SDG from seeds or the seed hulls has not been reported yet. Hence, a comparison of NPCE with other extraction methods on SDG extraction from flaxseed cakes is necessary. Lixiviating method (LM), ultrasonic assisted extraction (UAE), heat reflux extraction (HRE) and NPCE were compared for their performances of SDG extracting from flaxseed cakes at their optimized conditions (Table 3). The extraction yield of SDG by LM was lowest among that of NPCE, HRE and UAE, while UAE and NPCE got higher extraction purity. It is notable that although the extraction yields of SDG using HRE were almost the same as those using NPCE, the long-term heating at high temperature in HRE led to lots of impurities in the extract. For the extraction time and solvent usage, we found higher extraction efficiency by NPCE at 35 °C for 35 min as compared to those of UAE for 45 min at room temperature. Moreover, the equipment cost of UAE was higher [21]. Recently, microwave-assisted extraction of SDG in flaxseed has also been applied to save process time and improve the yield (11.70–21.45 mg/g DW) [15,33,34]. However, a higher solid/liquid ratio (21.9:1, 40:1 or 50:1) was utilized in microwave-assisted extraction. Additionally, enzymes such as β-glucuronidase, sulfatase and cellulase have been used for SDG release but without significant improvement [15,16,17]. In particular, high voltage electrical discharges were used for SDG extraction [18,19]. The electrical breakdown accompanied by high-amplitude pressure shock waves, bubble cavitation and liquid turbulence was used to accelerate extraction. However, the SDG yields (0.33–4.71 mg/g) were lower than those in the conventional extraction methods or NPCE (around 14 mg SDG/g flaxseed cakes) (Table 3). Furthermore, the design of NPCE equipment is simpler, and it can be easily scaled up in industrial production. Therefore, NPCE could be applied as a promising alternative for SDG extraction with several advantages including less time consumption, smaller requirement for solvents and relative low temperatures.

**Table 3:** Comparison of different extraction methods.

Method	Extraction Temperature	Extraction Time	Liquid/Solid Ratio (mL/g)	Pressure (MPa)	The yield of SDG (mg/g DW)	The purity of SDG (%)
HRE	90 °C	2 h	20:1	Atmospheric pressure	15.44 ± 0.06 ^a^	2.41 ± 0.19 ^a^
LM	35 °C	24 h	30:1	Atmospheric pressure	13.29 ± 0.69 ^b^	3.02 ± 0.31 ^a^
UAE	Room temperature	45 min	15:1	Atmospheric pressure	14.65 ± 0.53 ^a^	3.92 ± 0.34 ^b^
NPCE	35 °C	35 min	13:1	−0.04	15.61 ± 0.53 ^a^	3.87 ± 0.36 ^b^

^a,b^ shows the values are significantly different (*p* < 0.05) (*n* = 3).

## 3. Experimental Section

### 3.1. Reagents and Materials

SDG standard (98%, HPLC grade) was obtained from Shanghai Tauto Biotech Co., Ltd. (Shanghai, China). Other chemical reagents were supplied by Beijing Chemical Reagents Co. (Beijing, China). Deionised water was filtered by using PURELAB Ultra water-purification system from ELGA (ELGA LabWater, Lane End, UK).

### 3.2. Plant Material and Sample Preparation

**Figure 2:**
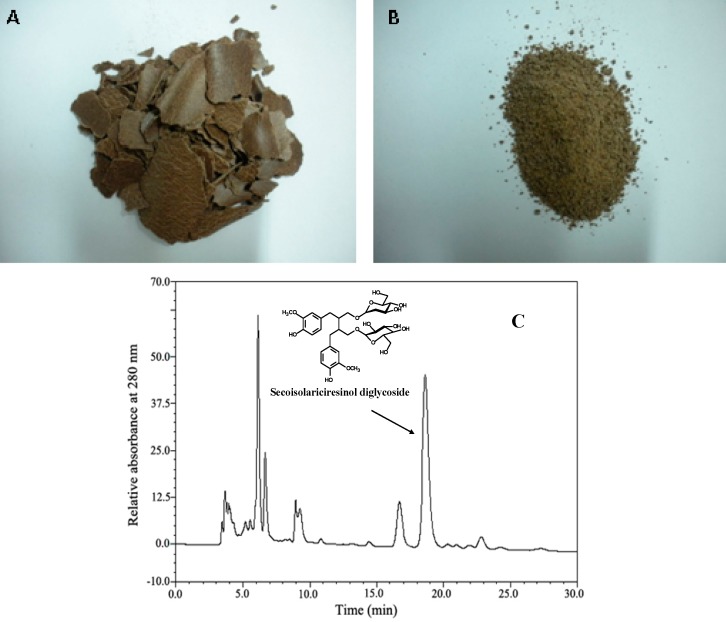
(**A**) The intact flaxseed cakes; (**B**) the crushed flaxseed cakes; (**C**) HPLC chromatograms of flaxseed cake extract at 280 nm.

Pressed flaxseed cakes (Figure 2A) were obtained after two cycles of mechanical expression of flax (*L. usitatissimum*, cultivar Yunnan YY-3) seeds from Institute of Industrial Crops in Yunnan Academy of Agricultural Sciences (Kunming, China). The initial dry matter (90%, *w*/*w*) of the samples was measured by desiccation at 105 °C until constant weight. The flaxseed cakes were ground in fine powders passing through 40 mesh. Crushed samples (Figure 2B) were stored at 4 °C for a maximum time of 24 h before further treatments.

### 3.3. NPCE Device

**Figure 3:**
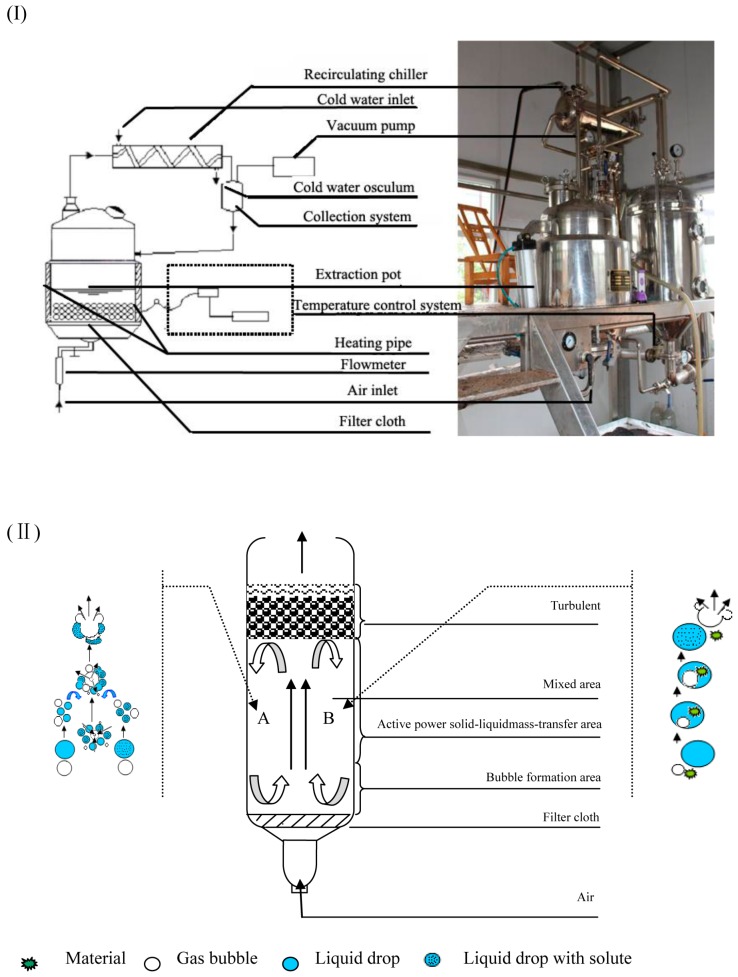
(**I**) Schematic diagram of the NPCE device; (**II**) the sketch map for the basic mechanism of the mass-transfer between liquid-liquid (A) and liquid-solid system (B).

NPCE equipment (CN2597047) was developed by the Key Laboratory of Forest Plant Ecology, Ministry of Education, Northeast Forestry University, Harbin, China [35]. The schematic diagram of NPCE device and the sketch map for the basic mechanism of the mass-transfer in the system were shown in Figure 3. It consists mainly of the extraction and collection pot. Materials and solvents are introduced into the extraction pot via the inlet. Subsequently, the negative pressure is generated by a vacuum pump, and air is introduced continuously through an inlet to a sieve plate at the bottom of the extraction pot, which is used to generate cavitations and filter the extraction solvent into the collection pot. The temperature in the extraction pot is controlled by a heating system. The volatilized solvent is cooled by the condenser. The tank volume of the extraction pot is 200 mL.

### 3.4. Extraction Procedures

#### 3.4.1. Lixiviating Method (LM)

After preliminary optimization experiments conducted according to the previous report [36], 2.50 g of pulverized sample were placed in conical flask with 75 mL of 1.39% (*w*/*v*) NaOH in 65% (*v*/*v*) ethanol solution. The extraction was performed using water bath in triplicate at optimized conditions (35 °C for 24 h) to obtain the best extraction efficiency.

#### 3.4.2. Heat Reflux Extraction (HRE)

The conventional HRE method was performed using a previously described method with slight modification [37]. The pulverized sample (2.50 g) was put into a round-bottom flask with 50 mL of 1.39% NaOH (*w*/*v*) in 65% (*v*/*v*) ethanol solution, then the flask was placed into a water bath and linked with a condenser at the joint of the flask. The extraction was performed at optimized conditions (90 °C for 2 h) with three replications.

#### 3.4.3. Ultrasonic Assisted Extraction (UAE)

2.50 g of pulverized sample were introduced into a conical flask with 37.5 mL of 1.39% (*w*/*v*) NaOH in 65% (*v*/*v*) ethanol solution. The extraction was performed in 40 kHz ultrasonic bath (Kunshan Ultrasonic Instrument, Kunshan, China, power 100 W) and carried out at optimized conditions (room temperature for 45 min) with three replicates [37].

#### 3.4.4. Negative-Pressure Cavitation Extraction (NPCE)

The sample (2.50 g) was put into the extraction pot. Then, the extraction parameters were set according to the Table 1. After being extracted, the solution was filtered and the residues were washed three times with 20 mL ethanol solution used in the corresponding run, then the filtered solution was combined together and adjusted to pH 7.0 with HCl solution (3 M). The total volume of the extraction solution was measured. At the same time, 10 mL was concentrated in order to dry, dissolved with 25 mL water and then kept at 2 °C for further analysis. The rest was then concentrated by rotary evaporator (Rotavapor R-215, BÜCHI, Flawil, Switzerland) and then freeze dried (Christ ALPHA1-2LD, Osterode, Germany) to get the weight of extraction. The yield of SDG and the extract purity were calculated by using the following equations:

(5) $$\text{The yield of SDG (mg/g)}=\frac{C\times V}{M}$$

(6) $$\text{The extract purity of SDG }(\%)=\frac{C\times V}{m\times 10}$$

where *C* is concentration of SDG (mg/mL), *V* is total volume of extraction solution (mL), *M* is the weight of dry material of flaxseed cakes (g), and *m* is the weight of the extracts (g).

### 3.5. The Experimental Design

#### 3.5.1. Fractional Factorial Design (FFD)

To approach the optimum region, FFD is the first step to estimate the main effects of complex factors [38]. The factors including extraction pressure (*X*_1_), temperature (*X*_2_), ethanol concentration (*X*_3_), amount of NaOH (*X*_4_), liquid/solid rate (*X*_5_), extraction time (*X*_6_), ventilation volume (*X*_7_) and soaking time (*X*_8_), were investigated using FFD in this experiment. In our design, the 2^9^ FFD with nine factors at two levels was applied with random run order. The statistics analysis was shown in Table 1.

#### 3.5.2. Central Composite Design (CCD)

CCD is applied to locate the true optimum parameters from a minimal number of possible experiments [39]. In this design, two critical influencing factors (*X*_4_-the amount of NaOH, *X*_5_-the liquid/solid ratio) were selected as independent variables in CCD based on the results of preliminary experiments (data not shown). Then, the standards of two factors design in 10 runs and two blocks of CCD were selected to determine the optimal conditions. In the CCD test, a total of 12 different experiments (four replicates of centre point) were optimized for fitting a quadratic polynomial regression equation model (Table 2):

(7) $$Y=\beta_{0}+\sum_{i=1}^{k}\beta_{i}X_{i}+\sum_{i=1}^{k}\beta_{ii}X_{i}X_{j}+\sum_{\begin{array}{l} i=1\\ i<j \end{array}}^{k-1}\sum_{j=2}^{k}\beta_{ij}X_{i}X_{j}$$

where *Y* was the predicted response, namely the dependent variable, representing the extraction yield of SDG. β_0_, β*_i_*, β*_ii_*, β*_ij_* were the regression coefficients for intercept, linearity, interaction and square, respectively. *X_i_* and *X_j_* were the coded independent variables, which influenced the response variable *Y* representing different levels of each factor.

The experimental data collected were processed by Statistica 6.0 software. The model significance and suitability was evaluated by the variance analysis (ANOVA) with 95% confidence level. A significance level of *p* < 0.05 was considered for each influential factor.

### 3.6. Analysis of SDG by HPLC

The yield and the extraction purity of SDG were determined by HPLC according to a previously described method [13]. Analyses were performed by using a DIONEX UltiMate^®^3000 UHPLC+ system (Dionex, Germering, Germany) equipped with an ultimate confirm the change 3000 pump and a DAD-3000 photodiode array detector. The system was run by using Chromelion software (version 6.80, Actuate, San Mateo, CA, USA). The separation was performed at room temperature on a DIONEX HPLC column RP-C_18_ (5 mm, 250 × 4.6 mm, Dionex, Germering, Germany). A solution of 10 μL of the sample was injected into HPLC for analysis. The mobile phase consisted of solvent A (methanol:acetonitrile = 9:1 *v*/*v*):solvent B (0.5% acetic acid in water, *v*/*v*) = 33:67 at a flow-rate of 0.8 mL/min. The detection was UV absorbance at 280 nm.

### 3.7. NPCE Pilot-Scale Experiment

The flaxseed cakes (2.00 kg) were put into an extraction pot of the pilot-scale equipment (Figure 3) and extracted under the condition of the optimum parameters obtained from CCD. Then, the extract was dried by rotary evaporator. The SDG yield and the purity in extracts were calculated and expressed as means ± standard deviation (SD) of triple separate experiments.

## 4. Conclusions

In the present study, the green NPCE process was optimized using RSM to present an efficient extraction of SDG from flaxseed cakes. The optimized conditions for SDG from flaxseed cakes using NPCE were: extraction pressure −0.04 MPa, extraction temperature 35 °C, ethanol concentration 65% (*v*/*v*), extraction time 35 min, liquid/solid ratio (mL/g) 13.16:1, the amount of NaOH 1.39% (*w*/*v*) and ventilation volume 90 L/h. Under these conditions, SDG extraction yield and purity was 16.25 mg/g, 3.86%, respectively. The response fitted models were verified and the experimental values suggested the reliability of the fitted models. Compared with the conventional extraction, NPCE offered a higher extraction yield of SDG and extraction purity. Moreover, it was proved that the optimized NPCE process was scalable by pilot-scale application. The NPCE was a good alternative for SDG extraction from flaxseed cakes in industrial production due to rapid extraction, high efficiency and high throughput.
